# Photoacoustic-Based Gas Sensing: A Review

**DOI:** 10.3390/s20092745

**Published:** 2020-05-11

**Authors:** Stefan Palzer

**Affiliations:** Department of Computer Science, Universidad Autónoma de Madrid, Francisco Tomás y Valiente 11, 28049 Madrid, Spain; stefan.palzer@uam.es; Tel.: +34-91-497-5720

**Keywords:** photoacoustics, gas sensing, absorption spectroscopy

## Abstract

The use of the photoacoustic effect to gauge the concentration of gases is an attractive alternative in the realm of optical detection methods. Even though the effect has been applied for gas sensing for almost a century, its potential for ultra-sensitive and miniaturized devices is still not fully explored. This review article revisits two fundamentally different setups commonly used to build photoacoustic-based gas sensors and presents some distinguished results in terms of sensitivity, ultra-low detection limits, and miniaturization. The review contrasts the two setups in terms of the respective possibilities to tune the selectivity, sensitivity, and potential for miniaturization.

## 1. Introduction

The photoacoustic effect has been described as early as the 19th century by several scientists [1,2,3,4,5], i.e., quite some time before the invention of the condenser transmitter [6,7] or other devices that would have allowed recording and quantifying of the parameters of a sound wave. However, the potential of converting light into sound was soon recognized and employed to detect gases. In particular, the possibility to gauge the light intensity using a gas-filled hermetically sealed cell has been explored in the context of improving the selectivity of the first so-called non-dispersive infrared spectrometer (NDIR) devices [8,9,10]. NDIR sensors at the time and still today show cross sensitivities to a large number of gas species because of the broad spectral response of solid-state-based light detectors. To overcome this drawback, first Schick in the 1920s and then Veingorow, Luft, and Lehrer realized the potential of using the target gas itself as the filter medium since it features near-identical spectral characteristics. This allows for maximizing the information content of the signal of the light transducer [11,12]. While working for the IG Farben constituent BASF, Ludwigshafen, Germany, Luft and Lehrer developed a measurement instrument based on this idea and applied for patent, which was granted in 1942 [13]. The so-called Ultrarotabsorptionsschreiber (URAS) (literal translation: ultra-red absorption writer) is still sold under this name and the rights and trademarks have since passed from BASF to Hartman&Braun, which currently is part of ABB, Zürich, Switzerland. 

Of course, research and development efforts related to the photoacoustic effect did not stop 70 years ago, including the establishment of theoretical foundations [14]. The historic evolution of technologies relying on the photoacoustic effect [11,15,16] as well as the closely related photothermal effect [17] are not the subject of this contribution. The application of the effect has branched out to various fields of application [18,19], including photoacoustic-based imaging, which is already covered in a number of reviews [20,21,22]. Because of the sheer vastness of the topic, this review focuses on the use of photoacoustics for gas sensing and in addition to previous related reviews [23,24,25,26]. Photoacoustics is a spectroscopic technique based on absorption of light and governed by the corresponding interactions of photons with matter. Therefore, this review will briefly revisit the relevant basic principles. As an absorption spectroscopy technique, it unites all the advantages of this approach, in particular the selectivity, sensitivity, and possibility for contactless sensing. Today, many research and development teams are working on improving the performance and devising new ways to employ the photoacoustic effect. This review on the topic can only hope to provide an overview over the most important aspects and results in this area. It is not intended to provide a complete list of all the literature available to date. 

## 2. Fundamentals of Photoacoustics

The prerequisite for photoacoustics is the absorption of light by particles and all considerations here are valid for the complete spectral range, even though the infrared spectral range may often seem the focus of attention. In terms of selectivity in gas sensing, this makes photoacoustics comparable to other absorption-based methods, most notably the so-called tunable diode laser absorption spectroscopy (TDLAS) [27,28] and non-dispersive infrared absorption spectroscopy (NDIR) [10,29], depending on the light source and setup used. Strong absorption features are linked with dipole-allowed transitions between electronic, vibrational, or rotational states as well as combinations thereof [30]. The strength of individual transitions between two states of a molecule or atom is summarized in a temperature (T)-, pressure (p)-, and frequency (ν)-dependent parameter, the so-called absorption cross section σ_T,p_ (ν). The line shape of σ_T,p_ (ν) is pressure and temperature dependent and for typically used light intensities, the saturation effects of a single absorption line [31] may be disregarded. The pressure-induced collisions of molecules/atoms give rise to a shortened lifetime of excited states, which in turn leads to a Lorentzian line shape in the frequency space. Likewise, temperature-induced random movement of an ensemble gives rise to Gaussian line shapes. In consequence, the relative importance of pressure and temperature for a given σ_T,p_ (ν) gives rise to the finally observed line shapes. 

In principle, various paths for relaxation of the excited system are possible upon absorption of a photon. Roughly, they may be separated into radiative and non-radiative processes or a combination thereof. For a strong photoacoustic signal, non-radiative relaxation processes should dominate over the re-emission of photons. In those cases where non-radiative processes are much quicker than radiative processes, most of the photon energy is converted into rovibrational and translational energy [18,32]. Ideally, the complete photon energy E_p_ = h v is converted into the internal and translational energy of the molecule. These systems tend to be well-suited for generating strong photoacoustic signals, since this will lead to local heating, which translates into an increase of temperature and pressure. Therefore, photoacoustic spectroscopy may be viewed as complementary to fluorescence spectroscopy. 

**Figure 1:**
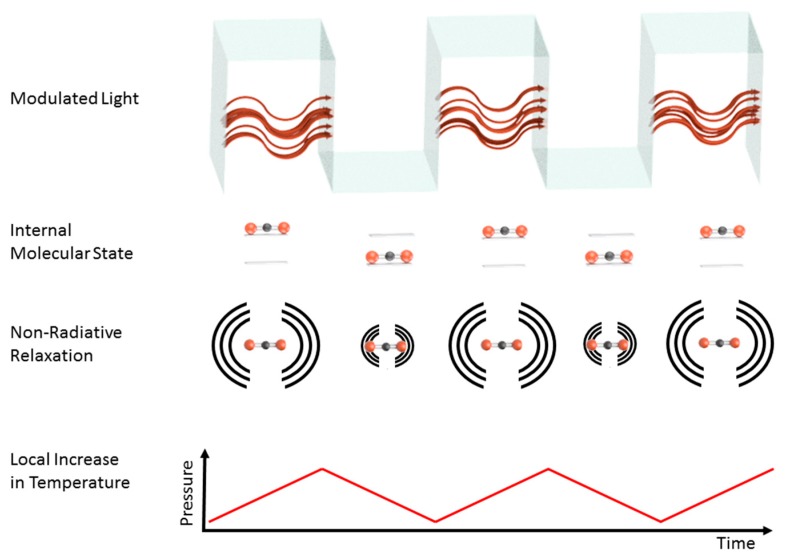
Simplified schematic depiction of the most relevant processes for photoacoustic signal generation. Intensity modulation of radiation excites internal molecular states. Due to non-radiative relaxation processes, part of the photon energy is converted into heat, leading to a local increase in temperature and pressure. Periodic light modulation thus leads to a pressure wave that may be detected using suitable transducer elements.

By modulating the light intensity, a periodic pressure variation can be generated, which in turn can readily be captured with a sound-detecting device like a microphone, a lever, or a tuning fork. This may be done by electronic means [33,34], opto-acoustic components [35], or mechanical choppers [36] in order to adjust the modulation frequency to the resonance frequency of the acoustic resonators. Figure 1 schematically depicts the processes involved in generating a sound wave. The type of modulation may vary, and the type of light source may limit the available soundwave excitation frequencies. When relying on using the light source’s driving current for intensity modulation, then thermal light sources are currently limited to frequencies on the order of 30 Hz [37]. Higher modulation frequencies using thermal sources may be achieved using a chopper. On the other hand, light-emitting diodes (LEDs) and diode lasers may easily be intensity modulated via their driving current. Frequencies up to several MHz can be achieved in theory. When using single-mode lasers, the use of wavelength modulation is a further option [34,38] to generate a photoacoustic signal. In any case, the intensity modulation frequency ν_M_ range is limited by fundamental considerations: On the one hand, it should be faster than the inverse of the molecular diffusion time 1/t_diff_, because otherwise no pressure wave may form. On the other hand, the modulation frequency has to be slower than the inverse of the molecular relaxation time 1/τ_R_, such that the system may react to changes [39]. 

However, instead of a classification in terms of light sources used, the discussion here will distinguish between two fundamentally different setups to use the photoacoustic effect for gas detection. For the remainder of the manuscript, setups are denoted as “indirect photoacoustics” when the sound-detecting device is not directly coupled with the analyte, i.e., the gas matrix to be analyzed. In contrast, in a “direct photoacoustic” setup, the sound generated by the analyte is detected while it is acoustically coupled with the medium to be analyzed. The potential in terms of sensitivity and miniaturization of the two approaches is governed by different parameters, which will become apparent in the corresponding discussion.

## 3. Direct vs. Indirect Setups

**Figure 2:**
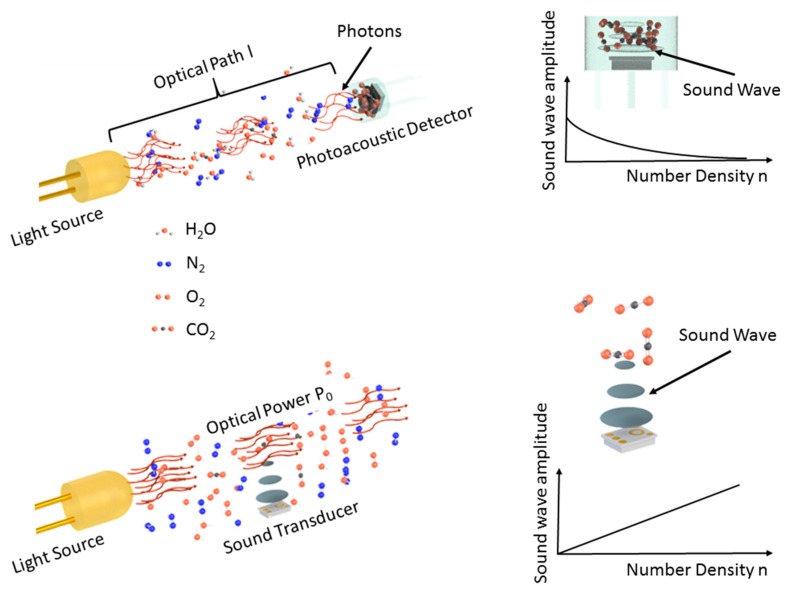
A schematic to compare the most basic direct (**bottom**) and indirect (**top**) photoacoustic spectroscopy setups. (**Top-left**) A photoacoustic detector is employed to determine the light intensity. The sensitivity is adjusted via the optical path length l and the detected acoustic signal diminished with an increasing number density of the gas to be detected (**top-right**). (**Bottom-left**) The sensitivity may be adjusted via the optical power P_0_ employed to excite the molecules. The acoustic signal increases with an increasing number density of the target gas (bottom-right).

Figure 2 schematically shows the detection of a representative trace gas (carbon dioxide—CO_2_), the main constituents of air (nitrogen—N_2_ and oxygen—O_2_), as well as often occurring and strongly varying humidity (water—H_2_O), which may also be the cause for cross-sensitivities. It depicts the two types of photoacoustic setups that can be distinguished depending on whether the gas mixture is to be analyzed is in direct contact with the sound detection device or not.

While this might seem a minor difference, it has important implications as to the modus operandi of systems and their respective potential in terms of miniaturization, sensitivity, and long-term stability. Many different and sophisticated devices have been developed using both types of setup. However, the inherent differences due to the different setups remain. Considering the application requirements is essential in opting for one way or another. Hopefully, this review and included discussion may act as a guide and orientation for the reader.

### 3.1. Indirect Photoacoustic (NDIR Setups)

**Figure 3:**
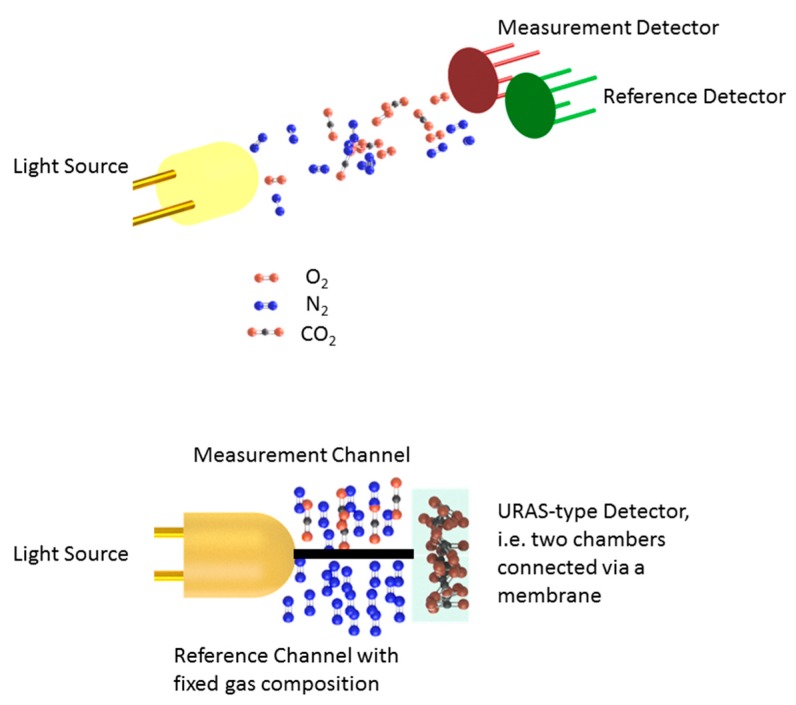
Comparison between a nowadays commonly used NDIR setup (**top**) and the URAS setup (**bottom**). (**Top**) Two detectors with different spectral response functions act as a measurement and reference channel, respectively. (**Bottom**) The URAS detector consists of two chambers filled with the gas to be detected and connected via a membrane. The optical path is divided into a reference part with a fixed gas composition and a measurement part containing the analyte.

The first commercially available photoacoustic-based gas detection devices were based on an NDIR-type setup, using a two-chamber system. Luft and Lehrer published [10] and patented [13] the so-called Ultrarotabsorptionsschreiben (URAS). The setup is depicted in Figure 3 and highlights several well-thought features, including an ingrained reference channel and a zero signal in the absence of the analyte. The basic idea of the setup is to use a hermetically sealed cell filled with the type of gas to be detected and equipped with a sound-detecting device to gauge the light absorption. This means that the setup is a non-dispersive absorption spectroscopy (NDIR) approach [29,40], where the attenuation of light in the optical path between the light source and light detector is used to infer the gas concentration. For trace gas sensing, one may assume that the Beer–Lambert–Bouguer law (also Beer–Lambert law) [41] is a valid approximation of the light attenuation:

(1) $$I\left(\nu ,\;l\right)=\;I\left(\nu ,0\right)e^{-\sigma_{p,T}\left(\nu \right)\cdot \;n\cdot \;l},$$

where I (ν, l) and I (ν, 0) is the light intensity at emission frequency ν after and before the optical path l, respectively, σ_p,T_ (ν) is the frequency-dependent absorption cross section of the gas species, and n is the number density. The very idea of NDIR spectroscopy is that no dispersive optical element is necessary to retrieve information regarding the gas concentration, hence the name. This in turn means, that the light detector performs an integration of all frequencies emitted by the light source within the response function, i.e., its output signal may be written as:

(2) $$A\;\left(n,\;\nu \right)\sim \int_{0}^{\infty }I\;\left(\nu ,l\right)\cdot D\left(\nu \right)\;d\nu ,$$

where D (ν) is the frequency-dependent responsivity of the detector. The dependence on the optical path length l means that the sensitivity and dynamic range of the setup can be tuned by that parameter. Saturation effects may occur if too many molecules absorb light prior to reaching the detector, which limits the dynamic range. The optical path length l is therefore the principal design tool to adjust the measurement range and resolution of NDIR devices. The degree of selectivity of this setup is subject to the spectral functions of both the light emitter as well as the detector, since the final signal is the result of a spectral convolution of the emitter and detector. In its most basic version, a spectrally broad-band [42] or narrow-band [43] thermal emitter may be combined with a broad band detector, such as a thermistor or photoresistor [44], which gives rise to a large number of cross-sensitivities to other gases. For that reason, spectral filters are oftentimes employed to limit the relevant spectral range to cover relevant absorption bands. Spectral filters can also be used to implement a reference channel that may account for fluctuations of the optical power emitted by the light source, e.g., a typical setup for an NDIR sensing device would employ two spectral filters, the first one a band pass for strong absorption bands of the gas to be detected and the second one a band pass where no or only very few molecules exhibit absorption [45]. 

Luft and Lehrer realized as early as the 1930s that the detector response function of standard broad-band detectors, such as photoresistors, introduce a larger number of sensitivities to other gas species and a suitable spectral filter may not always be feasible. This is especially relevant where many molecules show overlapping spectral features, e.g., in the 3 µm/3000 cm^−1^ spectral region, where many hydrocarbons exhibit strong absorption lines due to C-H stretch vibrations. They therefore came up with the idea of using the gas to be detected itself as a spectral filter. Additionally, they used the photoacoustic effect as a transducer mechanism to convert light power into an electrical signal. In order to realize a reference channel, two parallel optical paths of equal length are illuminated by the same light source. One path is filled with the gas mixture to be analyzed while the second path features an unchanging gas mixture containing a fixed concentration of the analyte. The detector devised in the URAS was (and is) made up of two hermetically sealed chambers filled with the gas to be detected. Both chambers are separated by a membrane that converts emerging pressure differences due to a non-zero difference in absorption along the optical path into an electronic signal. This means that a difference in the absorption along the respective optical paths results in a pressure difference in the URAS-type detector, serving as an indicator for the number density of the analyte. Since the excited sound wave amplitude is directly proportional to the light intensity, this allows for measurement of the concentration. The setup also ensures that there is no signal when no analyte is present. 

The development of the URAS-type sensor expanded from carbon monoxide (CO) sensing to include many other small molecules, including methane (CH_4_) and carbon dioxide (CO_2_) [46]. Additionally, more companies have since developed their own solutions based on similar principles. With the emergence of microsystems technology, efforts have begun to miniaturize the URAS setup [47,48,49]. To this end, simulations of the sensitivity function of standard NDIR and photoacoustic NDIR underscore the potential for miniaturization [50] and experimental data does support this [51]. 

The sensitivity is increased by roughly one order of magnitude as compared to NDIR devices using narrow-band interference filters depending on the spectral properties of the gas [52]. The reason is that only photons at frequencies coinciding with the absorption features of the target gas add to the signal for photoacoustic NDIR. Several versions of miniaturized photoacoustic NDIR have been demonstrated in recent years using thermal emitters, light-emitting diodes (LEDs), and lasers as light sources, and microelectromechanical systems (MEMS) microphones, quartz tuning forks (QTF) [53], or cantilevers [54] as sound transducers. Because most microphones offer a constant sensitivity over a large frequency range, the intensity modulation of the light source may be chosen quite freely, always taking into account that the photoacoustic signal amplitude decreases with an increasing modulation frequency 1/ν_MOD_ [14]. However, when using, e.g., a QTF, the photoacoustic signal has to coincide with the resonance of the system [55]. While this poses a restriction of the possible modulation frequencies, it also introduces a build-in narrow-band acoustic filter to reject ambient noise. Table 1 gives an overview of recent contributions that aim at miniaturizing URAS-type solutions, i.e., indirect photoacoustic spectroscopy setups, employing optical path lengths as short as 1 mm [56]. Most of these examples do not feature a reference channel, which is a shortcoming as compared to the original URAS-type devices because it hinders the construction of long-term stable devices that automatically correct for changes of the emission characteristics of the light source. One reason is that QTFs or MEMS microphones are often used as readily available standard transducers of sound to present proof-of-principle systems. Additionally, realizing URAS-type detectors in microsystems technology as well as system’s engineering is more challenging. Hence, it remains a task to come up with a configuration that realizes a reference channel when using photoacoustic detectors to build NDIR devices. One possibility is to build a system using a single light source featuring two identical detectors at different optical paths [51]. This way, the effective optical path length is reduced, but long-term changes in the light source may be corrected for. Alternatively, two photoacoustic detectors can be serially aligned, with one containing the target gas and the other filled with a gas that does not exhibit any or only partially overlapping spectral signatures. A correction for intensity fluctuations of the light source can be derived if both absorption spectra are within the emission spectrum of the light source, but only one reacts to the gas to be detected.

### 3.2. Direct Setups

**Figure 4:**
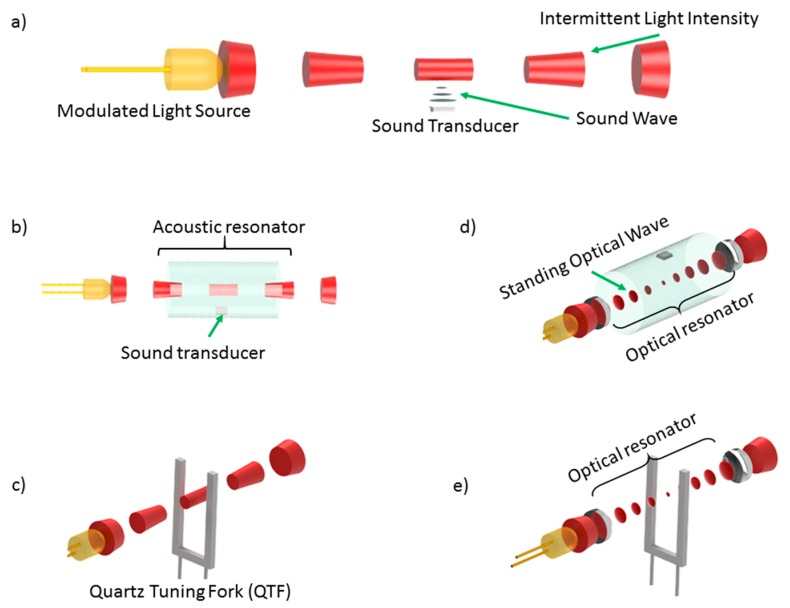
A schematic not-to-scale depiction of various basic setups for photoacoustic spectroscopy with different degrees of complexity. (**a**) The most basic setup is popular with several commercial manufacturers for its simplicity: A modulated light source generates an intermittent light beam, which in turn generates a photoacoustic signal. The amplitude of the sound wave is measured using a sound transducer, (**b**) Acoustic resonators are used to enhance the generated acoustic wave, (**c**) A quartz tuning fork (QTF) may serve as a low-cost narrow bandwidth resonator, (**d,e**) To increase the available optical power, an optical resonator or multi-pass cells may serve to multiply the photoacoustic signal.

A setup for direct photoacoustics differs fundamentally from NDIR, i.e., indirect setups, in its means of increasing sensitivity and achieving selectivity. While for indirect photoacoustics setups, sensitivity is mostly defined by the optical path length l, direct setups rely on increasing the optical power P_0_ to increase the acoustic signal generated. This becomes apparent when looking at the generation of a signal S [39]:

(3) $$S=C\cdot n\cdot \sigma_{p,T}\left(\upsilon \right)\cdot P_{0},$$

where C is a system-specific constant, n is the number density, and σ_p,T_ (ν) is the absorption cross section. In contrast to indirect setups, the selectivity is achieved via the spectral properties of the light source, including possible spectral filters. The implications of the signal generation on the gas response of sensing systems extends to the dynamic range because in direct setups, it is limited by the signal to noise ratio on the one hand, and the dynamic range of the sound transducer on the other hand. The latter may be extremely large, since, e.g., MEMS microphones typically have a 100-dB dynamic range, which is equivalent 5 orders of magnitude in the number density of the gas. Equation (3) shows that increasing the optical power will improve the system’s performance. Additionally, a reduction in acoustic noise and/or enhancement of the generated sound will help improve the signal to noise ratio, leading to ever more sensitive gas detectors, which is summarized in the parameter C. In fact, photoacoustic-based resonator-enhanced gas detection systems are among the most sensitive chemical sensors built to date, reaching limits of detection in the ppq (parts-per-quadrillion) range [65,66]. Especially since laser sources in the mid infrared range have become readily available [67,68], the field of laser-based photoacoustic spectroscopy has seen a stark increase in contributions. Nonetheless, the first laser-based photoacoustic-based gas sensing systems were demonstrated more than 50 years ago [69] and an ultra-sensitive device was reported as early as 1971 [70], highlighting the potential of the combination of a laser as the light source and the photoacoustic effect for chemical analysis and environmental studies [71]. Depending on the requirements of a specific measurement task, LEDs or thermal light sources might prove more suitable and in fact, many commercially available solutions use simple direct photoacoustic setups. Figure 4 summarizes the basic setups used to perform direct photoacoustic spectroscopy. It should be pointed out that a combination of acoustic resonator and, e.g., a QTF, is of course possible and has been demonstrated already [72]. Nonetheless, the choice for one approach or another will always depend on the system requirements as becomes clear from a comparison of microphone-based setups and a lever setup [73].

To optimize the photoacoustic signal and enable specific detection of a gas species, the use of laser sources plays a pivotal role in most laboratory setups. The high spectral density and beam quality allows for the design of ultra-sensitive devices and the use of enhancement strategies to increase the acoustic signal allows for the building of highly sensitivity and specific analyzers.

#### 3.2.1. Non-Resonator Based Setups

Depending on the requirements of a measurement task, the focus may lay on a simpler and less expensive setup rather than achieving ultra-sensitive and specific detection. Additionally, the usefulness of laser sources depends on the spectral functions of the analyte. One example is nitrogen dioxide (NO_2_), with an absorption spectrum in the blue/UV spectral range around 400 nm. The strongly overlapping electronic absorption features have a full width at half maximum (FWHM) width exceeding 100 nm [74,75] and the associated radiant decay gives rise to the typically brownish/red color of smog in polluted cities [76]. Hence, commercially available LEDs have a sufficiently narrow band to provide a reasonable degree of selectivity. Other examples include the CO_2_ absorption band near 4.2 µm, where few other gases absorb. In these cases, selectivity that is basically equal to standard NDIR is achieved using the most simple photoacoustic setup, i.e., a light source illuminating a volume and generating periodic pressure variations. A combined standard NDIR and direct photoacoustic device was presented in [77] and demonstrates a direct comparison. However, commercial examples of direct photoacoustic setups include the recently launched miniature sensors of Sensirion [78], and Infineon [79], the latter including a spectral filter around 4.2 µm to limit cross-sensitivities. The size of these systems is comparable to miniaturized indirect photoacoustic setups [52] but feature a lower degree of selectivity. Advanced Energy does offer the “Innova” series of systems based on the same principle but uses several spectral filters to excite soundwaves belonging to different gas molecules (formerly Lumasense) [80]. While the signal of these setups is proportional to the number density, they are also prone to drifts due to fluctuations of the emitted power as well as cross sensitivities towards other molecules with absorption lines overlapping the emission spectrum of the employed light source. However, current manufacturers prefer a simple setup in exchange for lower selectivity, and sensitivity. In order to improve both figures of merit, more complex setups are necessary, which often incorporate laser light sources. The potential of a non-resonant photoacoustic cell is highlighted in [66,81], where it is combined with a high-quality optical cavity to enhance the optical power to generate the photoacoustic signal. The limits of detection achieved in those works are in the ppt range. 

#### 3.2.2. Acoustic Resonator-Enhanced Photoacoustic Spectroscopy

From the very beginning of direct photoacoustic spectroscopy, the use of acoustic resonators has been a central engineering task and [32] provides a review of the basic concepts. An acoustic resonator may perform two tasks, namely the insulation from environmental acoustic noise and enhancement of the photoacoustic signal. Depending on the size of the system, different regimes of acoustic wave propagation and the formation of standing waves apply [82]. In any case, the most important figure of merit of this element is the Q-factor, which is an often-used figure of merit describing the quality of a resonator and the associated enhancement factor of a resonant wave. For acoustic resonators, the Q-factor may be expressed as [83]:

(4) $$Q=\frac{\upsilon_{0}}{\delta \upsilon },$$

where ν_0_ is the resonance frequency and δν the full-width half-maximum linewidth of the resonance. Consequently, the photoacoustic wave should match one of the resonance frequencies in order to achieve signal amplification. To build ultra-sensitive devices, a cancelation of ambient noise and parasitic signals, e.g., from windows, sophisticated and clever designs and modus operandi have been devised. Among the most widely used approaches is the use of so-called differential photoacoustic cells. The setup employs two equal acoustic resonators and compares the signals of the microphone placed in each. Two different methods to use this setup have been proposed: The more common method is to excite gas only in one of the resonators [84] but have a single buffer volume and windows for both resonators. This way, the noise detected by both microphones is equal and may be used to retrieve a signal only dependent on the light power and number density. Alternatively, the acoustic wave may be excited in both resonators but at a phase difference of π, i.e. 180°, which increases the signal by about a factor of 2 [85]. In any case, the problem of compensating for drifts in the light power of microphone sensitivity remains with these setups. Among the examples to improve the long-term stability and enable an automated compensation of drifts is the so-called differential mode excitation scheme [86]. It makes use of the detection of different modes of the resonator that carry different information regarding the gas concentration and noise, which ultimately leads to the retrieval of a purely gas-dependent signal using only a single resonator. A single resonator may also be used to detect various gases, e.g., by multiplexing the laser sources [87]. While many efforts are aimed at improving sensitivity, the long-standing goal of miniaturization of these setups to make them portable is being pursued as well. In terms of miniaturization, 3-D printers and silicon technology have been used to build simpler and smaller setups with diminished performances but at much lower cost and requiring less infrastructure [88,89,90,91,92,93,94].

Since the signal strength improves the performance in terms of a lower limit of detection and better resolution, the use of either multi-reflection cells or optical resonators has started to attract much interest over the years [95,96,97,98,99,100,101]. To this end, high-quality resonators may enhance the available optical power for signal generation by several orders of magnitude [102]. It is, however, important to note that changes in the optical alignment of mirror quality may lead to notable chances in the performance, which is why these parameters have to be precisely controlled. After all, as evident from Equation (3), any change in the optical power will lead to a change in the signal. The use of various enhancement techniques also makes the setup more complex, since several resonance conditions have to be fulfilled at once, including the absorption transition (central lasing frequency), the optical resonator (central lasing frequency), and the acoustic resonator (modulation frequency). In any case, it remains a fundamental issue that acoustic resonators are subject to changes in their Q-factor and resonance frequency resulting from changes in environmental parameters, including temperature [103,104], humidity [105], and the gas matrix itself [106], since the speed of sound depends on all these factors. Additionally, the collisional partner influences the sensing behavior as well [107]. This has to be taken into account when characterizing and deploying acoustic resonator-enhanced photoacoustic systems. Table 2 summarizes some systems that have employed acoustic resonators in the past years.

#### 3.2.3. Quartz-Enhanced and Cantilever Photoacoustic Absorption Spectroscopy

Low-cost reliable MEMS devices often allow for advances in miniaturization and improvement of performances in originally unforeseen applications. The last two decades have seen an increasing use of so-called quartz tuning forks (QTFs) [53] as well as micromachined levers [154] in sensing applications. Originally, these devices served, e.g., as a stable frequency reference [155] but since have found alternative applications, e.g., to read out atomic force microscopy (AFM) tips [156] as well as a sound transducer in photoacoustic setups. Levers as well as QTFs are governed by highly similar physical concepts from which their usefulness derives and are treated as equivalent for the remainder of this discussion. These readily available low-cost components feature a high Q factor and have been employed to gauge the photoacoustic sound wave amplitude for almost two decades now. When using a QTF, the setups are denominated as quartz-enhanced photoacoustic absorption spectroscopy (QEPAS) [157] and this name shall include lever-based setups here as well. As opposed to cavity-enhanced photoacoustic setups, QEPAS does not realize an acoustic resonator but the QTF is a resonator itself, i.e., the prongs of the fork (or the lever) resonate(s) when exited by a sound wave. Because of the high Q-factors of miniature low-cost tuning forks, it has become a central building block for small high-performance gas sensing devices. The higher the Q factor, the longer the time delay between two independent measurements. A number of reviews on QEPAS are available [158,159,160] and current research and development efforts are focused on reducing the size of systems, designing custom QTFs to lower the resonance frequency [72,161], as well as increasing the efficiency of the coupling sound to the prongs via excitation of the sound parallel to the prongs [162] as well as adding an acoustic resonator to a QEPAS setup [163]. 

The setup of QEPAS/lever systems is based on exciting the acoustic wave in-between the prongs of the fork or in proximity to a lever. Similar to acoustic resonators, QTFs also exhibit higher order vibrational modes that may be taken advantage of, e.g., by using a single QTF to detect various gases simultaneously [164,165]. To this end, a precise alignment of the laser beam is required in order to efficiently couple sound into the transducing device. The most common approaches include free-space optics using a focusing lens [166], tapered fiber setups, and fiber-coupled systems [167]. The read-out is usually based on a lock-in scheme in order to determine the oscillation amplitude at the resonance frequency using the 2f signal. Again, the laser power is crucial for the resulting signal and needs to be stabilized or at least measured with high precision in order to extract reliable number density readings. A change in the background gas composition may influence the signal and read-out strategies need to take this into account [168]. Additionally, environmental parameters influence the resonance frequency and bandwidth of the QTF. To overcome some of these issues, an alternative operational scheme was presented in [169] allowing for retrieval of the Q factor and resonance frequency of QTF with each measurement thus improving the calibration issues and long-term stability. 

Both lever-based as well as QEPAS systems have been used to achieve ultra-sensitive detection of various gas species with limits of detection in the ppt range [66,81,98,170,171] and in Table 3 several experimental setups are summarized.

## 4. Discussion and Conclusions

Direct and indirect photoacoustic systems both employ the photoacoustic effect for gas detection but differ fundamentally in their setups and working principles. When designing a gas sensing system, one therefore must be aware of the distinct factors that influence their respective performances in terms of selectivity, sensitivity, stability, robustness, and potential for miniaturization. 

For setups of indirect photoacoustic spectroscopy, the selectivity of the system is introduced via the gas filling of the hermetically sealed photoacoustic cell. An acoustic signal may only be generated when the emission spectrum of the light source and the absorption spectrum coincide. The gas filling hence acts as a near-ideal spectral filter and the gas to be detected can be chosen according to the requirements of the measurement task. On the other hand, selectivity in direct photoacoustic setups is entirely dependent on the spectral function of the light source, since an acoustic signal will be generated whenever a molecule absorbs radiation. For this reason, thermal emitters are usually combined with spectral filters in order to improve selectivity. Most setups use lasers as light sources because it enables the construction of specific gas detection systems.

In order to tailor the limit of detection, the sensitivity and the dynamic range of gas detection systems’ indirect setups use the optical path between the light source and detector. Using this knob, one may change these parameters, but a fundamental limit remains, namely that the sensor signal saturates for strong absorption along the optical path, thus limiting the achievable dynamic range. Additionally, increasing the optical path length, i.e., the sensitivity, will become more difficult the longer the optical path becomes. The use of acoustic resonators to improve the signal to noise ratio is of course possible, but little work has been done on this so far, other than using QTF as the sound transducer. In direct setups, the dynamic range is given by the sound-transducing device and hence can be improved by choosing or engineering suitable components. Both the limit of detection as well as the sensitivity may be engineered by the optical power used to excite the acoustic wave as well as enhancing the acoustic wave and improve its detection. 

Finally, the potential for miniaturization of both approaches is a more complex issue and depends very much on the application, in particular the required resolution and limit of detection. Indirect, i.e., photoacoustic NDIR, setups may be designed about one order of magnitude smaller as compared to standard NDIR setups while maintaining the same sensitivity and improved performance in terms of selectivity. However, a direct comparison with direct photoacoustics is more difficult, since the performance depends on the optical power available as well as the concentration range one has to monitor. When employing acoustic resonators, the size of the system cannot be reduced indefinitely, since an increase in the acoustic frequency leads to a decreasing amplitude. In terms of miniaturization, employing a QTF currently appears to be a promising candidate. However, it is difficult to combine with thermal light sources, because of the required high modulation frequency in the kHz range. At the same time, mid-infrared lasers are currently still expensive, meaning that miniaturization is feasible but not commercially reasonable. In any case, the technological choice depends on a multitude of parameters, including requirements on prize, system size, sensitivity, stability, selectivity, and calibration efforts. An example of this is the first commercially available miniaturized photoacoustic gas sensors, which employ a thermal light source at a low modulation frequency in combination with a spectral filter to detect carbon dioxide. Both the cross-sensitivities and the resolution are comparable to standard NDIR devices, thus not making full use of the potential of photoacoustics. The use of the photoacoustic effect in gas sensing has a long-standing history, but current research and development efforts show that the potential of the technique is far from fully exploited. Especially the use of MEMS technology in gas sensing systems will probably lead to further improvements in terms of the long-term stability, miniaturization, integration, and selectivity. The aim of this manuscript was to provide a quick introduction to the main areas of photoacoustic-based gas sensing and discussion of the different aspects of a specific implementation.

## Figures and Tables

**Table 1:** Photoacoustic NDIR demonstrations. Both the concentration range as well as the limit of detection are stated for ambient pressures of 1 bar. The values are stated either in part-per-million (ppm), parts-per-billion (ppb), or percent (%), whichever is most appropriate.

Light Source	Sound Transducer	Target Gas/Measurement Range	Concentration Range and Limit of Detection (LOD)	Citations
Thermal source	Microphone	Carbon dioxide (CO_2_)	0 %–100 % LOD not stated	[57]
			0 ppm–2500 ppm 8.69 ppm	[58]
		Carbon Monoxide (CO)	0 ppm–70 ppm LOD not stated	[59]
			0 ppm–2500 ppm 8.83 ppm	[58]
		Methane (CH_4_)	0 %–100 % LOD not stated	[57]
			0 ppm–2500 ppm 10.29 ppm	[58]
LED	Microphone	Carbon dioxide (CO_2_)	0 ppm–5000 ppm 100 ppm	[52]
			0 ppm–7000 ppm 345 ppm	[50]
			0 ppm–7000 ppm LOD not stated	[60]
			0 %–100 % 2,053 ppm	[56]
		Methane (CH_4_)	0 %–100 % LOD not stated	[61]
			0 %–100 % 5024 ppm	[56]
			0 %–6 % 2510 ppm	[51]
	QTF	Carbon dioxide (CO_2_)	0 %–100 % LOD not stated	[62]
		Methane (CH_4_)	0 %–100 % LOD not stated	[62]
Laser	Microphone	Methane (CH_4_)	0 ppm–1100 ppm LOD not stated	[63]
	Cantilever	Ethylene (C_2_H_4_)	0 ppm–1000 ppm 10 ppb	[64]

**Table 2:** An excerpt of past acoustic resonator-enhanced photoacoustic demonstrations. All references have used a microphone as sound transducer. Abbreviations LD: Laser Diode, QCL: Quantum Cascade Laser, OPO: Optical Parametric Oscillator, LED: Light-Emitting Diode.

Target Gas (es)	Light Source	Reference
Nitrogen Dioxide (NO_2_)	LD	[108,109,110,111,112]
	Multimode LD	[113]
	LED	[114,115,116,117,118]
Nitric Oxide (N_2_O)	QCL	[112,119]
	OPO	[120]
Nitrogen oxide (NO)	QCL	[121]
	LD	[122]
Ammonia (NH_3_)	QCL	[123,124,125]
	CO_2_ laser	[126,127]
	LD	[128]
Carbon Dioxide (CO_2_)	QCL	[129]
	CO_2_ laser	[130]
	LD	[131,132,133,134]
	OPO	[135]
Hydrogel Sulfide (H_2_S)	QCL	[136]
	CO_2_ Laser	[130]
	LD	[137,138,139]
Water (H_2_O)	QCL	[125,140]
	LD	[133,134,141]
	LED	[142]
Acetylene (C_2_H_2_)	CO_2_ Laser	[143]
	LD	[87,144,145]
Methane (CH_4_)	QCL	[146,147,148]
	LD	[87,133,146,149]
	OPO	[150]
Ozone (O_3_)	QCL	[151]
	quadrupled Nd:YAG	[152]
Hydrogen Chloride (HCl)	LD	[149,153]
Ethylene (C_2_H_4_)	CO_2_ Laser	[98]

**Table 3:** An excerpt of past QEPAS/cantilever demonstrations.

Target Gas(es)	Setup	Reference
Nitrogen Dioxide (NO_2_)	QTF with acoustic resonator	[172,173]
	QTF using LED	[174]
Nitrous Oxide (N_2_O)	QTF with optical cavity	[164,175,176]
Nitric oxide (NO)	QTF using laser	[177]
Ammonia (NH_3_)	QTF using laser	[178,179]
Carbon Dioxide (CO_2_)	QTF using laser	[178,180,181,182]
	QTF with optical cavity	[183]
	LED with cantilever	[184]
	Laser with cantilever	[185]
Hydrogel Sulfide (H_2_S)	QTF using laser	[179,186,187,188]
Water (H_2_O)	QTF using laser	[169,189,190]
Acetylene (C_2_H_2_)	QTF using laser	[191,192,193]
Methane (CH_4_)	QTF using laser	[164,169,175,194]
Ozone (O_3_)	QTF using laser	[195]
Hydrogen Chloride (HCl)	QTF using laser	[196]
Ethylene (C_2_H_4_)	QTF using laser	[197,198,199,200]
